# Does dietary nitrate boost the effects of caloric restriction on brain health? Potential physiological mechanisms and implications for future research

**DOI:** 10.1186/s12986-023-00766-9

**Published:** 2023-10-25

**Authors:** Mushari Alharbi, Blossom CM Stephan, Oliver M Shannon, Mario Siervo

**Affiliations:** 1 School of Life Sciences, The University of Nottingham Medical School, Queen’s Medical Centre, Nottingham, NG7 2UH UK; 2 Department of Clinical Biochemistry, Faculty of Medicine, King Abdulaziz University, Jeddah, 22252 Saudi Arabia; 3 Curtin Dementia Centre of Excellence, EnAble Institute, Curtin University, Kent Street, Bentley, WA 6102 Australia; 4 Human Nutrition Research Centre, Population Health Sciences Institute, Newcastle University, Newcastle upon Tyne, NE2 4HH UK

**Keywords:** Dietary nitrate, Nitric oxide, Caloric restriction, Brain health, Cognitive function, Endothelial function, Dementia

## Abstract

Dementia is a highly prevalent and costly disease characterised by deterioration of cognitive and physical capacity due to changes in brain function and structure. Given the absence of effective treatment options for dementia, dietary and other lifestyle approaches have been advocated as potential strategies to reduce the burden of this condition. Maintaining an optimal nutritional status is vital for the preservation of brain function and structure. Several studies have recognised the significant role of nutritional factors to protect and enhance metabolic, cerebrovascular, and neurocognitive functions. Caloric restriction (CR) positively impacts on brain function via a modulation of mitochondrial efficiency, endothelial function, neuro-inflammatory, antioxidant and autophagy responses. Dietary nitrate, which serves as a substrate for the ubiquitous gasotransmitter nitric oxide (NO), has been identified as a promising nutritional intervention that could have an important role in improving vascular and metabolic brain regulation by affecting oxidative metabolism, ROS production, and endothelial and neuronal integrity. Only one study has recently tested the combined effects of both interventions and showed preliminary, positive outcomes cognitive function. This paper explores the potential synergistic effects of a nutritional strategy based on the co-administration of CR and a high-nitrate diet as a potential and more effective (than either intervention alone) strategy to protect brain health and reduce dementia risk.

## Introduction

Dementia is a progressive, incurable neurodegenerative disease leading to significant alterations of brain structure and function, resulting in cognitive decline, physical impairment, and changes in behaviour [1, 2]. Worldwide, more than 50 million people had dementia in 2020 and this figure is predicted to increase three-fold by 2050 [2, 3]. Cases are not distributed equally across the globe with most (> 60%) cases living in low- and middle-income countries where resources, research and policy focused on dementia is scarce [2, 4, 5]. The increasing number of older adults aged 65 years and over represents one of the major drivers of the growing number of dementia cases globally and the large proportion of dementia cases are expected to occur in very old individuals (≥ 80 years) [3, 5, 6].

Dementia has a multifactorial pathogenesis, and is linked to a plethora of modifiable and non-modifiable risk factors including for example increased age, female gender, genetics (e.g., Apolipoprotein e4 allele status), nutrition (poor diet), lifestyle (e.g., smoking, physical activity), socioeconomic status (e.g., deprivation), low education, and poor cardio-metabolic health status (e.g., hypertension, diabetes and obesity) [7]. With no cure, the maintenance of a healthy physical and cognitive trajectory across the life course is an international public health priority to reduce the projected number of dementia cases impacting not only the individual, but also society.

Numerous observational and experimental studies have investigated the links between nutrition and the brain health ranging from testing associations and effects of dietary patterns (i.e., Mediterranean Diet, Dietary Approach to Stop Hypertension (DASH) Diet, to single foods (i.e., green leafy vegetables, oily fish) and nutrients (i.e., minerals, vitamins, phytochemicals) provided alone or in combination [8–10]. Caloric restriction (CR) and, more recently, an increased dietary nitrate consumption have been linked independently with several health benefits including anti-ageing effects and improvements of brain health and cognitive performance [8–10]. Some of the key biological mechanisms underpinning the benefits of CR and dietary nitrate on brain physiology involve the modulation of oxidative stress [11–13], inflammation [14], mitochondrial function [11, 12], insulin [15, 16], and nitric oxide signalling and autophagy [17–19]. This opinion paper provides a brief overview of key nutritional factors that may influence brain health, and it proposes a physiological rationale for the synergistic effects of combined CR and dietary nitrate interventions on brain health as an effective strategy for dementia risk reduction and prevention.

### Ageing, obesity and vascular dysfunction: the dementia risk triad

Ageing is linked to a progressive decline of vascular, metabolic, and neurocognitive functions [20]. Some of the mechanisms underpinning these functional declines include reduced metabolic efficiency, decreased anti-inflammatory responses, elevated production of reactive oxygen species (ROS), and declined nitric oxide (NO) production [1, 21–24]. A progressive loss of synaptic connectivity, neuronal plasticity and accumulation of aberrant native proteins (Beta-Amyloid, Tau-Protein, Lewy-Bodies) are key features of the ageing process [22]. In most individuals, these changes do not result in clinical manifestation of cognitive impairment or dementia [22]. However, if functional and structural damages become more extensive and overcome compensatory mechanisms, cognitive dysfunction may accelerate and lead to the onset of clinical dementia [22]. For a detailed review of pathogenetic hallmarks of ageing and dementia risk, see Hou et al. [20].

Obesity is causally linked to various chronic conditions including diabetes, hypertension, coronary heart disease, and cancer [25, 26]. Obesity has also been associated with an accelerated cognitive decline across the life course including impairments in global cognition, logical memory, delayed recall, and verbal fluency [25]. Mid-life obesity is a key risk factor for the onset of late-life dementia [25–27]. Obesity also showed an increased risk of atrophy in grey and white matter regions (frontal, temporal and occipital cortices, thalamus, hippocampus, and midbrain) and is linked to a reduction of regional blood flow in the pre-frontal cortex [26]. Excess adiposity has been linked to a decreased whole-body NO production and endothelial dysfunction (could be a result of a reduction in NOS activity [28]), which may affect neuro-vascular coupling, blood-brain barrier (BBB) permeability and reduced cerebral blood flow (CBF) [25, 27]. Obesity-related vascular dysfunction significantly impacts brain function and increases the risk across the various dementia sub-types as cerebrovascular dysfunction represents a common pathogenetic feature [1, 24]. A reduction of nitric oxide (NO) bioavailability has been linked to hypertension and cerebral hypoperfusion, which are closely linked to the occurrence of major events in the brain such as cerebral ischemia and stroke [24, 29, 30].

### Nutrition and brain health

Maintaining brain functions requires an optimal supply of energy and nutrients. The brain is an energy-demanding organ (20% of the total body energy production), heavily relying on the oxidative metabolism of carbohydrates and fat [31, 32]. Glucose and ketone bodies are the primary sources of energy for the brain to drive ATP production, preserve neuronal and glial cellular integrity and ensure the efficiency of neurotransmission [33]. Polyunsaturated fatty acids (omega-3), vitamins B (1, 6, 9, and 12), D, E, and C, minerals (iron, copper, calcium, and zinc), and other nutrients with antioxidant properties (i.e., polyphenols, dietary nitrates) may have a crucial role in the preservation of cerebrovascular and cognitive functions by regulating synaptic transmission, membrane fluidity, endothelial function, and neurotransmitter and signal-transduction pathways [8–10].

Unhealthy dietary patterns, sedentary lifestyle, social isolation, low educational attainment, smoking, and alcohol addiction are common risk factors for cardiovascular disease and cognitive impairment [2, 21]. In the last decade, greater emphasis has been given to multi-dimensional approaches to dementia prevention, including testing the effects of healthy dietary patterns and providing multiple sources of protective nutrients [34–38]. The Mediterranean diet (MED) and Dietary Approaches to Stop Hypertension (DASH) are examples of dietary patterns, which have been linked to a reduction in cardiovascular and dementia risk in observational and intervention studies [34, 35]. Morris et al. have amalgamated the key features of the two dietary patterns to propose the MIND diet (MED + DASH), which essentially promotes a high consumption of plant-based products (similar to the MED, but with a particular emphasis on increasing the intake of berries and green leafy vegetables) to reduce dementia risk [37]. These dietary patterns emphasize the consumption of fruits, vegetables, whole grains, nuts, seeds, and healthy fats. [37, 39, 40] and encourage a controlled energy intake to match or reduce levels below an individual’s energy requirements (CR). They are rich in protective nutrients including fibre, mono- and poly-unsaturated fatty acids, vitamins, antioxidants, and other nutrients such as polyphenols or dietary nitrate that can positively influence vascular, metabolic, and cognitive functions [30, 41–49]. Dietary nitrate may represent a crucial health-enhancing element within plant-based dietary regimens [50, 51]. Hord et al. [52] conducted an estimation indicating that the DASH diet has the potential to deliver as much as 1200 mg/day of dietary nitrate. This is in comparison to the typical daily intake of approximately 110 mg/day found in the general population [53]. In randomized clinical trials, a common dosage of dietary nitrate involves supplementation of around 600 mg/day, achievable through the consumption of two bottles of concentrated high-nitrate beetroot juice [51]. CR strategies and dietary nitrate may therefore represent potential effective nutritional strategies to prevent both endothelial and cognitive dysfunction, thus, reducing the risk of dementia.

### Caloric restriction

#### Current evidence

CR aims to reduce the daily caloric intake without causing malnutrition to enhance physical and mental health [54]. CR has been linked to an increase in lifespan across various species and a decrease in age-related morbidity and mortality including rodents, primates, and humans [21, 54–62]. In addition, CR enhances the neuro-inflammatory responses [14] and lowers the occurrence of oxidative damage by improving mitochondrial efficiency [11, 63, 64], with a reduction of white matter loss [62], improved cerebral blood flow [21, 56] in several brain regions [11, 64], and enhanced cognitive function [14]. Although much of the evidence for a salutary effect of CR is derived from animal model studies, some human investigations have also identified promising effects of CR on markers of cardiometabolic/brain health.

Forty-nine healthy overweight and obese older adults were randomised to a three-month CR intervention which significantly improved memory, insulin, glucose, and C Reactive Protein compared to high PUFA and a control diet [15]. Nevertheless, not all CR studies have reported beneficial effects [65–67]. This could be related to the heterogeneous methods employed, including differences in the CR protocol (e.g., different caloric intake and intervention duration) and study cohort (e.g., animal, and human populations with different ages, sex, and health status). For example, a 6-month randomised trial tested the interactive effects of CR and exercise in forty-eight participants but no significant improvement in cognition was found [67]. In young rats, a two-month CR intervention had an adverse effect on the brain, decreasing neurogenesis and spatial learning assessed using the Morris water maze [68]. On the other hand, a more extended CR intervention (ten months) with older mice showed an improvement in spatial learning [69]. The characteristics of some of the key studies, identified by a non-systematic search of human randomised clinical trials (RCTs) on PubMed, that have investigated the effects of CR on brain health (cognitive function and CBF) are reported in Table 1.

**Table 1:** Key studies investigating the effects of caloric restriction on brain health in humans

Reference	Population	Study design	Measurements	Intervention	Main finding
**Witte et al. 2009** [15]	Healthy overweight elderly. n = 49 (M/F = 21/29). Age = 60.5 ± 7.6 SD. BMI = 28 ± 3.7 SD.	Parallel RCT.	Memory performance, BP, CRP, TNF- α, BDNF, glucose, insulin and lipid profile.	Duration: three months. Three groups: 1. CR (30% reduction in EI) n = 19. 2. Increase UFAs (20%) n = 20. 3. Control n = 10.	CR increases memory score significantly (20%; p < 0.001), and it has a significant inverse association with insulin, glucose and CRP among the high compliance subjects. No significant difference in UFAs and control.
**Zotova et al. 2015** [114]	Arterial hypertension (AH) and cerebral ischemia (CI) patients. n = 42 into two arms: 1. CR (M/F = 6/16), age = 54.4 ± 2.4 SD. 2. Antihypertensive drugs (M/F = 8/12), age = 55.6 ± 1 SD.	Parallel controlled clinical trial.	Cognitive function, cerebral haemodynamic (Doppler ultrasound), QoL, glucose, and lipid profile.	Duration: six months. Two groups: 1. CR n = 22. Level of CR not reported. 2. Antihypertensive therapy (ACE inhibitors, thiazide diuretics), neurometabolic drugs, drugs that improve cerebral hemodynamics) n = 20.	CR significantly improves the cognitive function, cerebral haemodynamic and QoL in both AH and CI compared to the second group and baseline.
**Prehn et al. 2017** [115]	Healthy postmenopausal obese women. n = 37. Age = 61 ± 5 SD. BMI = 34.9 ± 4 SD.	Parallel RCT.	Memory performance, cognitive function, fMRI (BOLD; oxygenation metabolism), physical activity, BP and glucose.	Duration: three months (CR) + one month of sustained weight loss (Isocaloric diet). Two groups: 1. CR (formula-diet 800 kcal/d) n = 19. 2. Control n = 18.	Improved recognition memory significantly and grey matter in the CR group compared to the control at the second time point (after the three months CR); p < 0.05, and it returned to non-significant at the endpoint, but it remained higher in CR.
**Kim et al. 2020** [116]	Healthy adults with central obesity. n = 43. Age = 52.8 ± 2 SD. BMI = 31.4 ± 5.1 SD.	Parallel RCT.	Memory performance, cognitive function, cardiometabolic, BP, glucose and lipid profile	Duration: one month. Two groups: 1. Continuous CR (500 kcal reduction), n = 22. 2. Intermittent CR (5:2 pattern; consuming 600 kcal for two consecutives days), n = 21.	Both groups enhanced the pattern separation significantly (p < 0.0005), but the intermittent CR group were significantly lower in recognition memory (p < 0.007).
**Leclerc et al. 2020** [117]	Healthy non-obese adults. n = 220. Age = 21–50 (males), 21–47 (female). BMI = 22–28.	Parallel RCT (part of CALERIE study).	Working memory, cognitive function, mood state, sleep quality and energy expenditure.	Duration: two years. Two groups: 1. CR (25% reduction). 2. Control.	CR improve working memory significantly compared to the control at second (12 months) and third (24 months) time points (p < 0.001).
**Teong et al. 2021** [118]	Healthy overweight and obese women. n = 46. Age = 50 ± 9 SD. BMI = 32.9 ± 4.4 SD.	Parallel RCT (secondary analysis).	Cognitive function, mood state, sleep quality and QoL.	Duration: two months. Two groups: 1. CR (30% reduction in EI) n = 24. 2. Intermittent fasting (IF; 30% reduction in EI) n = 22.	Both groups increase cognitive function significantly (CR; p < 0.006, IF; p < 0.03). There was no significant difference in the other measurement, except that weight loss was significant in the IF group (p < 0.001).

**Note**: The list is not comprehensive (e.g., generated using systematic review methodology), but provides a selection of key studies that have contributed to the field. **Key**: BDNF, brain-derived neurotrophic factor; BOLD, blood oxygenation level-dependent, BP, blood pressure; CRP, c-reactive protein; fMRI, functional magnetic resonance imaging; M/F, males/females; QoL, quality of life; RCT, randomised controlled trial; SD, standard deviation; TNF- α, tumour necrosis factor-alpha; UFAs, unsaturated fatty acids, EI, energy intake

#### Key molecular mechanisms

The main molecular pathways linking CR to the improvement of endothelial and cognitive functions involve sirtuins (SIRT; proteins family), protein kinase B (Akt), AMP-activated protein kinase (AMPK), mechanistic target of rapamycin (mTOR), autophagy and NO [17]. Sirtuins could be upregulated by various stressors such as energy reduction (CR); when activated and overexpressed, sirtuin catalyses NAD-dependent deacetylase, which has been found to be associated with longevity [58, 59]. In addition, SIRT1 could act as an antioxidant that influences several protein regulations (such as p53, Ku/Bax and FOXO) to resist the stress-induced damage, reduce apoptosis and protect neurons [58, 70]. Additionally, SIRT1 is involved in various metabolic pathways linked to adiposity (PPARγ downregulation), insulin, glucose, and lipid metabolism (PGC-1α and LXRα deacetylation, and UCP2 expression) [58, 70]. Sirtuins have a significant role in enhancing NO bioavailability by activating endothelial nitric oxide synthase (eNOS), directly or indirectly, through the activation of the AMPK pathway [29, 71, 72]. CR-induced Akt phosphorylation through the insulin-PI3K-Akt signalling pathway is important for cell growth and resilience, and synaptogenesis [73], which could enhance vascular [71] and cognitive [74] functions. The activation of AMPK, SIRT1, and Akt during CR plays a crucial role in regulating endothelial function via the Ca2+/calmodulin-dependent kinase II (CaMKII) stimulation, which upregulates eNOS and leads to an increased NO synthesis [75]. CR stimulates autophagy [69, 76] and downregulates hippocampal mTOR, which acts as a neuroprotector by reducing neuronal apoptosis [77]. Results from experimental models also call for a more cautious interpretation of the current evidence as an excessive NO production, associated with the activation of iNOS, may be related to the pathogenesis of neurodegenerative diseases such as Parkinson’s and Alzheimer’s disease [78, 79]. The existence of an optimal range of NO production is established as both high and low production rates have been linked to abnormal pathogenetic processes [80]. However, an increased NO production, achieved via the stimulation of the nitrate-nitrite-NO pathway and still maintained within an optimal range, has been consistently associated with positive effects on several physiological functions [81].

### Dietary nitrate

#### Current evidence

Inorganic or dietary nitrate is a water-soluble polyatomic ion which can be found in various food sources; particularly green leafy and root vegetables (e.g., beetroot) [52, 82]. Dietary nitrate may be an effective nutritional intervention for improving vascular and metabolic health via an increased NO production produced in the nitrate-nitrite-NO pathway [83–85]. Dietary nitrate supplementation may reduce the risk of cognitive decline by improving neuronal metabolism and CBF, with effects on several domains including decision-making and memory [86–88]. A systematic review and meta-analysis of 16 RCTs, including 254 participants, assessing the impact of dietary nitrate on blood pressure, showed a significant reduction in systolic (-4.4 mm Hg; p < 0.001) and diastolic (-1.1 mm Hg; p < 0.06) BP, and a significant inverse association between the daily nitrate intake and systolic BP (p < 0.05) [44].

A double-blind, crossover RCT showed that a 3-day dietary nitrate supplementation in healthy young males improved brain oxygen metabolism and CBF [89]. Two RCTs recruited healthy young adults (age = 24.4 ± 5.7 SD) and ischemic overweight old patients (age = 67.4 ± 10.2 SD) and both found a significant enhancement of CBF after one-week dietary nitrate supplementation [90, 91]. A single administration of nitrate-rich beetroot juice to healthy young (age range 18 to 27) participants significantly improved cognitive function and CBF measured by Near-Infrared Spectroscopy (NIRS) at rest and during cognitive stimulation [45]. These effects could be explained by several mechanisms such as improvement of endothelial function, neurovascular coupling and cerebral autoregulation due to an increased NO bioavailability. Despite these promising findings, not all studies have reported a beneficial effect of dietary nitrate supplementation on cognitive function/cerebral blood flow. A systematic review and meta-analysis of twenty-two RCTs investigating the impact of dietary nitrate on cognitive function (n = 13 studies, total participants = 297) and CBF (n = 9 studies, total participants = 163), found no significant effects of dietary nitrate supplementation on cognitive function or CBF. However, most studies were of short duration (time range 90 min to 3 days) and included mostly young (mean age 22.6) non-obese healthy participants with the exception of one study testing effects of sodium nitrite in older adults (aged 50 to 79) for longer duration (10-weeks) [88]. Also, a more recent RCT not included in the meta-analysis (which addressed many of the limitations of earlier studies in this area) reported no significant effects of a 13-week dietary nitrate supplementation on cognitive function and CBF measured by NIRS in overweight and obese older adults (n = 62, age range 60 to 75 years) [92]. However, they found that the moderate and low dosages could have a significant improvement on systolic BP (low; p = 0.03, and moderate; p = 0.04) and microvascular perfusion (p = 0.02 for both arms in both outcomes) when compared to high and placebo groups, without significant difference between the moderate and low doses [93]. Hence, the duration and dosage need to be considered when evaluating the current literature. The lack of convincing evidence, the short duration of the studies to justify changes in cognition, and limited sensitivity of some methods to measure CBF and microvascular perfusion certainly call for more robust study designs and adoption of deep-phenotyping approaches to evaluate the effects of dietary nitrate on brain functions.

Dietary nitrate is closely linked with dietary antioxidants and oxidative metabolism. The ingestion of compounds with anti-oxidant properties such as ascorbic acid, vitamin E or phenolic compounds (i.e., quercetin, resveratrol) can enhance the generation of NO by promoting a greater conversion of nitrate into nitrite in the gastric acidic lumen and/or by reducing the presence of ROS capable of quenching and inactivating both nitrite and NO [94–96]. Supplementation of dietary nitrate after acute hyperglycaemia in old obese adults decreased levels of two independent markers of oxidative stress significantly when compared to the placebo (3-nitrotyrosine; mitochondrial superoxide production in peripheral blood mononuclear cells (PBMCs)) [12]. Larsen et al. [97] demonstrated in humans that the dietary nitrate administration for 3 days improved oxidative phosphorylation efficiency (P/O ratio) and induced a decrease in state 4 respiration in skeletal muscle, which mechanistically was linked to a reduced expression of a protein involved in proton conductance (ATP/ADP translocase). The same group subsequently demonstrated in an animal model of renal and cardiovascular diseases that dietary nitrate was able to decrease oxidative stress markers in plasma (malondialdehyde) and urine (Class VI F2-isoprostanes and 8-hydroxy-2-deoxyguanosine) [98]. An increased dietary nitrate intake induced upregulation of catalase, superoxide dismutase, glutathione peroxidase, mitofusin 2 and PGC1α in PBMCs in patients with metabolic syndrome [99]. Nevertheless, no significant effects of dietary nitrate supplementation were found on markers of oxidative stress (i.e., malondialdehyde, mitochondrial superoxide, 8-isoprostane) in other studies [100–102], which clearly emphasizes the need for further basic and translational research in this area.

Some of the key human RCTs, identified by a non-systematic search of PubMed, that investigated the effects of dietary nitrate on brain health (cognition and CBF) are described in Table 2.

**Table 2:** Findings from key studies reporting the effects of dietary nitrate on brain health in humans

Reference	Population	Study design	Measurements	Intervention	Main finding
**Aamand et al. 2013** [89]	Healthy young men. n = 18. Age = 25 ± 0.9 SD. Weight = 77 kg ± 1.5 SD.	Double-blind, crossover, placebo-controlled RCT.	fMRI (BOLD; oxygenation metabolism and ASL; CBF), nitrate, nitrite, BP, pulse oximetry, expired CO_2,_	Duration: three days for each intervention with a washout period of 9–11 days. Two groups: 1. Start with dietary nitrate (NaNO_3_^−^; saline solution), n = 9, followed by Placebo (NaCl; saline solution). 2. The opposite of the first group, n = 9.	Dietary nitrate decrease haemodynamic lag significantly (p < 0.005), which associate significantly with NO_3_^−^ concentration (p < 0.05). In addition, it improves the BOLD amplitude significantly (3-way ANOVA; p < 0.05), without significant association with NO_3_^−^ concentration. Moreover, a significant correlation between the lag and amplitude (p < 0.005). Furthermore, dietary nitrate increases the NO_3_^−^ concentration significantly (p < 0.001) despite the intervention order, but it were not significant for the NO_2_^−^ in both intervention. However, there was no significant difference in the other measurement.
**Kelly et al. 2013** [119]	Healthy old adults. n = 12 (M/F = 6/6), two were excluded. Age (M/F) = 64 ± 4 SD / 63 ± 2 SD. BMI (M/F) = 23.1/25.1.	Double-blind, crossover, placebo-controlled RCT.	Nitrite, BP, physiological and cognitive examinations.	Duration: two and half days for each intervention with a washout period of three days. Two groups: 1. Start with dietary nitrate (high-nitrate beetroot juice; 2 × 70 ml/d), n = 6, followed by placebo (depleted-nitrate beetroot juice; 2 × 70 ml/d). 2. The opposite of the first group, n = 6.	Dietary nitrate enhanced NO_2_^−^ concentration significantly (p < 0.01) when compared to placebo and baseline. In addition, it significantly reduced systolic and diastolic BP when compared to baseline (p < 0.01) and placebo (p < 0.05). However, there was no significant difference in the cognitive function between nitrate intake and placebo or baseline.
**Wightman et al. 2015** [45]	Healthy adults. n = 40 (M/F = 12/28). Age = 21. BMI = 24.36	Double-blind, parallel, placebo-control RCT.	Cognitive function (COMPASS), CBF (oxyhaemoglobin and deoxyhaemoglobin by NIRS), BP, nitrite.	Duration: single high dose, over three portions, separated by 10 min, measurements performed after one and half hours from the first portion for one hour approximately during cognitive tasks. Two groups: 1. Beetroot Juice 450 ml (nitrate-rich), n = 20. 2. Placebo (nitrate-depleted), n = 20.	Inorganic nitrate increase nitrite significantly (p < 0.01). In addition, it significantly increases CBF at the beginning of the tasks (total Hb; P < 0.05) and decreases it significantly afterwards (p < 0.01) compared to placebo. However, there was no significant difference between the groups regarding the deoxy Hb. Moreover, it improves cognitive function (p < 0.01). However, there was no significant difference on BP.
**Fan et al. 2019** [90]	Healthy young adults. n = 17 (M/F = 10/7). Age = 24.4 ± 5.7 SD. BMI = 23.2 ± 2.1 SD.	Single-blind, crossover, placebo-controlled RCT.	CBF, cerebrovascular CO_2_ activity, cerebral autoregulation (BP and MCAv), nitrate and nitrite.	Duration: one week for the baseline assessment plus one week for each intervention with a washout period of one week. Two groups: 1. Start with dietary nitrate (NaNO_3_^−^; 3 capsules/d), followed by placebo (microcrystalline cellulose; 3 capsules/d) 2. The opposite of the first group	Dietary nitrate enhanced NO_3_^−^and NO_2_^−^concentrations significantly compared to baseline and placebo (p < 0.002), without a significant difference between males and females. In addition, it improved the arterial stiffness significantly compared to baseline and placebo (p < 0.008). Moreover, it improved significantly cerebral autoregulation compared to placebo but not to baseline in males but not females (p < 0.025). Furthermore, it improved MCAv-CO_2_ only in males compared to placebo (p < 0.014). However, there was no significant difference in BP or cerebrovascular haemodynamic
**Fan et al. 2020** [91]	Transient ischemic attack overweight patients (TIA). n = 26. Age = 67.4 ± 10.2 SD. BMI = 27.9 ± 6.4 SD.	Single-blind, parallel, placebo-controlled RCT.	Cerebrovascular function (BP and CBF), cerebrovascular CO_2_ activity, cerebral autoregulation, nitrate and nitrite.	Duration: one week. Two groups: 1. Dietary nitrate (NaNO_3_^−^; 3 capsules/d), n = 13. 2. Placebo (microcrystalline cellulose; 3 capsules/d), n = 13.	Dietary nitrate significantly increased concentrations of NO_3_^−^ (p < 0.001) and NO_2_^−^ (p < 0.004) compared to placebo and baseline (p < 0.001). Additionally, placebo was not significant for both NO_3_^−^and NO_2_^−^ compared to baseline. In addition, it decreased the BP significantly compared to placebo and baseline (p < 0.05). Moreover, it improves MCAv variability (p < 0.018) and cerebral autoregulation (p < 0.045) compared to placebo. However, there was no significant difference in cerebral haemodynamics compared to placebo.
**Babateen et al. 2022** [92]	Healthy overweight and obese adult. n = 62 (M/F = 24/38). Age = 66.3 ± 3.7 SD. BMI = 30.3 ± 3.7 SD.	Single-blind, parallel, placebo-controlled pilot RCT.	CBF (oxyhaemoglobin and deoxyhaemoglobin by NIRS) and cognitive function (COMPASS).	Duration: 13 weeks. Four groups: 1. High dietary nitrate (high-nitrate beetroot juice; 2 × 70 ml/d) n = 16. 2. Moderate dietary nitrate (high-nitrate beetroot juice; 70 ml/d) n = 17. 3. Low dietary nitrate (high-nitrate beetroot juice; 70 ml/alternate days) n = 14. 4. Placebo (depleted-nitrate beetroot juice; 70 ml/ alternate days) n = 15.	There was no significant difference between the groups and baseline in terms of CBF and cognitive function.
**Alharbi et al. 2023** [13]	Healthy overweight and obese adult. n = 29 (M/F = 7/22). Age = 61.3 ± 5.9. SD. BMI = 34.5 ± 5.8 SD.	Open-label, parallel, pilot RCT.	Body composition, REE, resting BP, endothelial activity, microvascular perfusion (Laser Doppler), cognitive function, hand-grip strength, physical activity, and oxidative stress biomarker.	Duration: 2 weeks. Two groups: 1. CR (40% reduction in EI) plus dietary nitrate (high-nitrate beetroot juice; 70 ml/d) n = 15. 2. CR alone (40% reduction in EI) n = 14.	There was significant improvement of systolic BP (p = 0.06), microvascular perfusion (p = 0.03), endothelial activity (p = 0.02), cognitive function (p = 0.01), and oxidative stress biomarker (p = 0.02) among CR + dietary nitrate group compared to CR alone. In addition, there was significant inversed correlation between oxidative stress biomarker and microvascular perfusion (r=-0.53, p = 0.003).

**Note**: This is a selection of key studies that have contributed to the field and they were not identified by a systematic search. **Key**: ASL, arterial spin labelling; BOLD, blood oxygenation level-dependent; BP, blood pressure; CBF, cerebral blood flow; CR, caloric restriction; fMRI, functional magnetic resonance imaging; MCAv, Middle Cerebral Artery Velocity; M/F, males/females; NIRS, near-infrared spectroscopy; REE, resting energy expenditure; RCT, randomised controlled trial; SD, standard deviation, EI, energy intake

**Molecular Mechanisms**: Dietary nitrate could improve brain health via increased NO production. Dietary nitrate requires reduction by oral microbiota (e.g., Firmicutes, Proteobacteria, Actinobacteria, and Bacteroidetes) [103] into nitrite [83, 104–106] or can be converted into nitrite more slowly via mammalian nitrate reductases [107]. Nitrite is then reduced to bioactive NO either in the stomach (acidosis) or in the circulation after absorption by the intestine (especially in hypoxia), and this requires reduction by enzymes (e.g., XOR: xanthine-oxidoreductase), haemoglobin, myoglobin polyphenols, ascorbate or protons [83, 104–106]. NO plays an essential role in regulating mitochondrial efficiency, immune and vascular smooth muscle cells (VSMC), and neuronal metabolism. NO can have direct or indirect effects; the former is possibly the most significant which involves the NO-cGMP pathway via sGC activation, an increase of cGMP production, which impacts the vascular smooth muscle cells (VSMC) and platelet function through cGMP-dependent protein kinase (PKG) production [83, 104–106]. PKG activates the myosin light-chain phosphatase (MLCP) and vasodilator-stimulated Phosphoprotein (VASP) that are linked to vasodilation, anticoagulation and reduced VSMC proliferation [83, 104–106]. NO can also influence mitochondrial metabolism by binding with the cytochrome c oxidase (62, 74, 76), enhancing the efficiency of respiratory chain and reducing ROS production via a competing interaction of reactive nitrogen species (RNSs) with complex 1 of the respiratory chain [18]. NO may improve pre-synaptic neurotransmission by facilitating the opening of the voltage-gated Ca^+ 2^ channels (VGCCs). This mechanism facilitates the transfer of Ca^+ 2^ to the post-synaptic (anterograde signalling) space via the NMDA receptor (activated by glutamate) to bind with Calmodulin (CaM), leading to the activation of nNOS and generation of NO [108, 109]. The locally produced NO exerts retrograde signalling to the pre-synaptic space, and this mechanism appears to be important for the consolidation of memory and learning (long-term potentiation; LTP) [108, 109]. Dietary nitrate could induce autophagy by PPAR expression, SIRT3 and AMPK activation [18, 19]. Studies testing the effects of dietary nitrate on glucose and insulin metabolism in animals and humans have produced mixed findings [12, 16, 110, 111]. The putative effects of dietary nitrate on glucose uptake may be linked to an increased generation of NO via the XOR pathway, consequent activation of PKG signalling and increased expression of glucose transporters (GLUT-1, GLUT-4 and HK-2) [110]. However, the exact mechanisms underpinning the effects of nitrate-nitrite-NO on brain metabolism are still largely unknown.

### Does dietary nitrate boost the effects of caloric restriction on brain health?

**Fig. 1:**
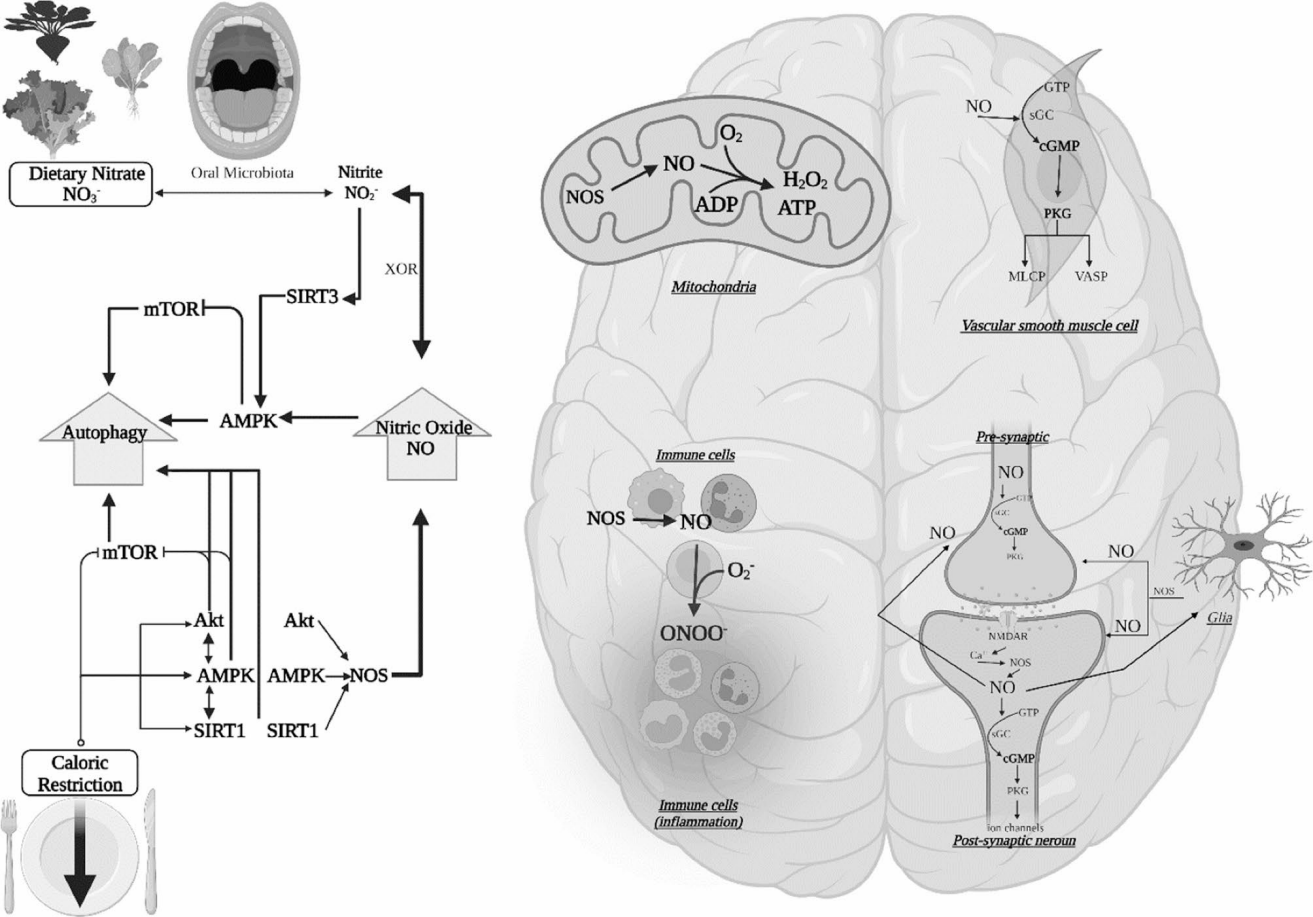
Synergistic effects of dietary nitrate and caloric restriction on brain health. Both interventions could induce NO bioavailability through the nitrate/nitrite/NO pathway or Akt, AMPK and SIRT1 pathways. NO would increase mitochondrial efficiency by reducing ROS and inducing ATP from oxygen and ADP. In addition, NO would improve the endothelial function by interacting with sGC to convert GTP into cGMP, which activates PKG leading to MLCP (smooth muscle relaxation) and VASP (platelet aggregation inhibitor) activation. Moreover, NO could modulate inflammation that acts as pro-inflammatory when it reacts with O2- (from uncoupled mitochondria) to form ONOO-. Furthermore, NO could enhance neurotransmission through activation of the antero-and retrograding signalling, which facilitates Ca + transferal. CR and dietary nitrate have several pathways that could increase the autophagy process by mTOR inhibition, Akt, AMPK, and SIRT activation. **Key**: AMPK, adenosine monophosphate-activated protein kinase; Akt, protein kinase B; cGMP, cyclic guanosine monophosphate; GTP, guanosine triphosphate; MLCP, myosin light-chain phosphatase; mTOR, mechanistic target of rapamycin; PKG, protein kinase G; sGC, soluble guanylate cyclase; SIRT1 and SIRT3, sirtuin; VASP, vasodilator-stimulated phosphoprotein; XOR, xanthine-oxidoreductase

It is possible that CR and dietary nitrate could have synergistic/additive effects on brain health via their effects on common mechanistic pathways involving regulating mitochondrial, metabolic, immune, endothelial and neuronal functions. Figure 1 provides a schematic representation of the putative mechanistic pathways. As described in the CR and dietary nitrate sections on molecular mechanisms, both interventions could influence mitochondria efficiency by enhancing the efficiency of respiratory chain, reducing ROS generation and increasing ATP yield. CR positively impacts on macronutrient oxidative metabolism via activation of SIRT1, Akt, AMPK and NO pathways; similarly, dietary nitrate enhances the NO bioavailability with a potential impact on glucose and lipid metabolism via increased GLUT-1, GLUT-2, GLUT-4, PPAR-alpha and AMPK expression. These combined mechanisms could potentiate the effects of the single interventions on maintaining a healthy ageing trajectory and reducing the risk of chronic metabolic and neurodegenerative diseases. Autophagy is a critical process for maintaining cell function via the coordinated removal and recycling of damaged and dysfunctional molecules [112, 113]. An increase in autophagy activity has been linked to both interventions via mTOR inhibition (by CR) [69, 76], and increased PPAR expression and AMPK activation (by dietary nitrate) [18, 19]. CR and dietary nitrate could have a synergistic effect on NO production via the activation of different pathways influencing both the enzymatic and non-enzymatic synthesis NO pathways including for example the activation of the SIRT, Akt and AMPK pathways. Alharbi, et al. (2023) showed for the first time that the combination of dietary nitrate with CR for two weeks among middle-aged and older adults with overweight and obesity improved microvascular perfusion (p = 0.03), cognitive function (TMT-B; p = 0.01), and reduced urinary 8-isoprostanes (p = 0.02) compared to CR alone [13]. The derived synergism of the two interventions on the proposed mechanisms may provide an effective strategy to minimise age-related cognitive decline and reduce dementia risk.

## Conclusions

Against the background of an ageing society and an impending increase in dementia cases, there is an urgent need to identify strategies to maintain healthy active ageing, including a specific focus on brain ageing. Dietary interventions have the potential to reduce the risk of age-related diseases including cardiometabolic and neurodegenerative conditions. Some, but not all, previous investigations have suggested that CR and dietary nitrate can have beneficial effects on metabolic, vascular, and cognitive functions. However, this evidence is typically characterised by small sample sizes, short-duration interventions, and a preponderance of young, healthy participants. Moreover, studies have applied a variety of different cognitive tools and imaging methods, contributing to heterogeneous results. In this paper, we advocate for a synergism between CR and dietary nitrate which could provide a feasible and more effective nutritional strategy (than either intervention alone) to improve cardio-metabolic and brain health. Currently, only one study has tested this hypothesis, which showed preliminary benefits of a combined CR and dietary nitrate intervention on endothelial and cognitive function. We identify plausible mechanistic pathways through which combined CR and dietary nitrate could improve cardio-metabolic and brain health. As a first step towards investigating the potential additive/synergistic effect of these two dietary strategies, we advocate prospective epidemiological studies to investigate the association between CR and dietary nitrate, alone and combined, with cognitive impairment and dementia in healthy and in ‘at risk’ populations. Such investigations could provide potential proof-of-concept, which could be further explored in randomised controlled trials focusing on feasibility, acceptability, and efficacy. Given the absence of effective treatments for dementia, the identification of novel dietary (and other lifestyle) approaches to reduce societal burden of this condition are greatly needed.

## Data Availability

Not applicable.

## Abbreviations

CR: Caloric Restriction
NO: Nitric Oxide
CBF: Cerebral Blood Flow
NOS: Nitric Oxide Synthase
eNOS: Endothelial NOS
nNOS: Neuronal NOS
iNOS: Inducible NOS
ROS: Reactive Oxygen Species
BMI: Body Mass Index
